# Supplementary material for: Unsupervised multi-source domain adaptation with no observable source data
Source: PLoS One. 2021 Jul 9;16(7):e0253415. doi: 10.1371/journal.pone.0253415 (PMC8270218; doi:10.1371/journal.pone.0253415)
Supplement: Attachment — Submitted filename: rebuttal_letter.pdf [file pone.0253415.s001.pdf]

Unsupervised Multi-Source Domain Adaptation with No Observable Source Data

Hyunsik Jeon, Seongmin Lee, and U Kang

We would like to thank the reviewers for their high quality reviews and constructive comments. Below, we summarize how we addressed the reviewers' requirements.

1. Reviewer 1.
   - **(R1-1)** Please highlight the contribution clearly in the introduction.
     - **(A1-1)** The contents of the contribution list have been revised to clearly highlight the contributions (lines 38-40 and lines 43-44 in introduction section).
   - **(R1-2)** This paper lacks in novelty of the proposed approach. The author should highlight the contribution clearly in the introduction and provide a comparison note with existing studies.
     - **(A1-2)** We revised the contribution list (lines 38-40 and lines 43-44 in introduction section) and provided a comparison note with the competitors in the introduction (lines 29-34 in introduction section). The existing studies for unsupervised multi-source domain adaptation (UMDA) assume that source data are observable and they train the adaptation networks to align manifolds of source and target domains. Thus, the existing methods are not applicable to our setting where source data are not observable. On the other hand, DEMS trains the adaptation networks while regulating the results of the source classifiers (lines 67-72 in related work section).
   - **(R1-3)** Some Paragraphs in the paper can be merged and some long paragraphs can be split into two.
     - **(A1-3)** We reviewed the overall manuscript to reorganize the paragraphs in it. Especially, we itemized the main ideas which were in one long paragraph into individual items (lines 104-126 in proposed method section).
   - **(R1-4)** The quality of the figures can be improved more. Figures should be eye-catching. It will enhance the interest of the reader.
     - **(A1-4)** We improved Figure 3, which previously looked complicated, to be eye-catching.
   - **(R1-5)** What are the computational resources reported in the state-of-the-art for the same purpose?
     - **(A1-5)** We implemented all the codes using PyTorch and trained all networks including DEMS and competitors using RTX 2080 Ti (lines 288-289 in experiments section).
   - **(R1-6)** Please cite each equation and clearly explain its terms.
     - **(A1-6)** We cited all equations in the manuscript and clearly explained the terms (lines 143, 147, 164, 179, 203, 204, and 213 in experiments section).
   - **(R1-7)** Math work should be written math mode.
     - **(A1-7)** We already wrote the math works by math mode. If there is anything we missed, please let us know and we will fix it.
   - **(R1-8)** What are the evaluations used for the verification of results?
     - **(A1-8)** We used validation set and trained DEMS until the loss $\mathcal{L}_{total}$ of the validation set is the lowest. Each experiment is performed 5 times with different random seeds and we reported the standard deviation along with the averaged accuracy. We added such description in lines 232-233 in proposed method section and lines 278-281 in experiments section.
   - **(R1-9)** Clearly highlight the terms used in the algorithm and explain them in the text.
     - **(A1-9)** We summarized the training algorithm of DEMS and explained the process in algorithm section (lines 226-240 in proposed method section).
   - **(R1-10)** Authors should add the most recent reference: 1) DARE-SEP: A Hybrid Approach of Distance Aware Residual Energy-Efficient SEP for WSN, IEEE Transactions on Green Communications and Networking, and 2) Interference Mitigation in D2D Communication Underlying

Cellular Networks: Towards Green Energy, Computers, Materials & Continua.
  - **(A1-10)** We added the two references (lines 273-276 in experiments section).

2. Reviewer 2.
   - **(R2-1)** What are the limitations of the existing works that motivated the current research?
     - **(A2-1)** Previous works have focused on unsupervised multi-source domain adaptation (UMDA) where source data are accessible. Thus, they trained the adaptation networks to align manifolds between source and target domains using the source and the target data. However, source data are not easily accessible in practical scenarios although source classifiers are readily accessible. Hence, we are motivated to develop a method to train adaptation networks using the source classifiers and the target data without using the source data. We added such discussion in lines 67-72 in related works section.
   - **(R2-2)** Summarize the key findings from the related works in the form of a table.
     - **(A2-2)** We summarized the key findings from the related works and compared them with our proposed method in Table 3 (lines 61-62 in related works section).
   - **(R2-3)** Some of the recent works such as the following on DNN/ML can be discussed in the paper: "An Effective Feature Engineering for DNN using Hybrid PCA-GWO for Intrusion Detection in IoMT Architecture", and "An optimal transportation routing approach using GIS-based dynamic traffic flows"
     - **(A2-3)** We added the two references (lines 273-276 in experiments section).
   - **(R2-4)** Present computational complexity of the proposed approach.
     - **(A2-4)** We added the computational complexity of DEMS (lines 236-240 in proposed method section).
   - **(R2-5)** Compare the current work with recent state-of-the-art.
     - **(A2-5)** The data-free UMDA is a novel problem since there were no previous approaches which can work without the source data. Nevertheless, we introduced several baselines in Table 1 and explained them in the introduction (lines 29-34 in introduction section).
   - **(R2-6)** Discuss about the limitations of the current work in conclusion.
     - **(A2-6)** We included the limitations of the work in conclusion section (lines 351-354 in conclusion section).
